# Eurasian beavers in Central Italy: perceptions in the local community

**DOI:** 10.1007/s00114-023-01860-x

**Published:** 2023-06-22

**Authors:** Andrea Viviano, Roger E. Auster, Giuseppe Mazza, Alessandro Lagrotteria, Chiara Pucci, Davide Senserini, Roisin Campbell-Palmer, Robert Needham, Davide Curci, Emiliano Mori

**Affiliations:** 1 Consiglio Nazionale Delle Ricerche – Istituto di Ricerca sugli Ecosistemi Terrestri – Via Madonna del Piano 10, 50019 Sesto Fiorentino (FI), Italy; 2 University of Exeter, Amory Building, Rennes Drive, Streatham Campus, Exeter, EX4 4RJ UK; 3 CREA Research Centre for Plant Protection and Certification (CREA-DC), Cascine del Riccio, Via Lanciola 12/a, 50125 Florence, Italy; 4 National Biodiversity Future Center, 90133 Palermo, Italy; 5 Università del Piemonte Orientale, Via G. Ferraris 107, 13100 Vercelli, Italy; 6 Independent Researcher, Str. Di Pilli 1, 53035 Siena, Italy; 7 Independent Researcher, Loc. Defizio, 58036 Grosseto, Italy; 8 Beaver Trust, 61 Bridge Street, Kington, HR5 3DJ UK; 9 Università Degli Studi Di Torino, Via Giuseppe Verdi, 8, 10124 Torino, Italy

**Keywords:** *Castor fiber*, Central Italy, Human dimensions, *Myocastor coypus*, Social perception, Reintroduction

## Abstract

According to the IUCN guidelines, wildlife reintroduction should consider any impacts on humans within feasibility assessments. Eurasian beavers *Castor fiber* are recovering across their native range, due to protection laws and reintroductions. In Central Italy, a self-sustaining, naturalised population of Eurasian beavers has been identified in the last five years. A questionnaire to measure whether and how citizens in the local area perceive the presence of the beaver was administered to 1114 respondents. We observed a comprehensive awareness of the presence of the beaver in Italy and a high ability to distinguish it from non-native coypus *Myocastor coypus* (92.3%). We also recorded a general high knowledge of issues related to the presence of the beaver (i.e., potential effects on indigenous biodiversity). The majority (65.5%) of the surveyed population was in favour of reintroducing the beaver in Central Italy, and only 1.2% was firmly against it. The majority of interviewed people was against the removal of beavers from Central Italy (65.8%), whereas only 3.7% was in favor, citing fears of perceived impacts on the river, crops, and fish populations.

**Supplementary Information:**

The online version contains supplementary material available at 10.1007/s00114-023-01860-x.

## Introduction

Global land use and climatic change, together with human-mediated animal translocations, have deeply altered worldwide biogeographical patterns (Higgins 2007; Young 2014).

Several species are naturally expanding their range, e.g., through the re-colonisation of landscapes abandoned by humans, recovery of forest habitats, or in response to climate change, with animals and plants migrating towards northern latitudes and higher altitudes (e.g., the Savi’s pipistrelle *Hypsugo savii*, the golden jackal *Canis aureus*, and the crested porcupine *Hystrix cristata* in Europe, respectively: Ancillotto et al. 2018; Spassov and Acosta-Pankov 2019; Mori et al. 2021a). In recent decades, increased environmental awareness and ethical motivations linked to the current sixth global biodiversity crisis have also triggered a number of reintroduction events and rewilding initiatives throughout Europe and North America (Halley and Rosell 2002; Schmitz et al. 2015; Schepers and Jepson 2016; Mueller et al. 2020). In detail, reintroductions are one form of animal translocation- where individuals of a species are released in areas where the species was present in historical times, but has since become extinct, usually following standard protocols and feasibility analyses (Seddon et al. 2007; IUCN 2013; Robert et al. 2015). Conversely, “rewilding” is the practice of returning areas of land to a wild state, which can include the release of animal species which no longer occur in these areas (Noguès-Bravo et al. 2016).

Reintroduction and rewilding initiatives often seek to reverse human impacts on wildlife, but they may also pose challenges. For example, the activities of reintroduced species may conflict with human activities, pose zoonosis risks, or there may be conflict between people about the species or approaches to wildlife management (Tattoni et al. 2017; Moseby et al. 2018; Thulin and Röcklinsberg 2020; Auster et al. 2020a, 2021).

Thus, alongside stakeholder engagement, assessment of social perceptions in the human population is an increasingly recognised pivotal step to help the success of reintroduction programs, as well as of any other wildlife management action including removal of alien species (Sharp et al. 2011; Hiroyasu et al. 2019; Kapitza et al. 2019). Particularly, Auster et al. (2022a) defined “renewed coexistence” as the coexistence and linked challenges between humans and reintroduced species, to encourage approaches to reintroduction that seek to foster sustainable coexistence with reintroduced species.

In this context, the Eurasian beaver *Castor fiber* is an emblematic species. In Medieval times, this species occurred throughout the Palearctic, in all suitable habitat types (Halley et al. 2021). Eurasian beavers are large semi-aquatic rodents that live in freshwater habitats. The species underwent a severe population decline due to intense hunting for fur, meat, and demand for *castoreum*, reducing the species to a small number in a few refugia (Campbell-Palmer et al. 2016). In the twenty-first century, beavers have recovered throughout most of their historical range, resulting from a combination of natural spread and human-led reintroduction efforts (Halley et al. 2021). Beavers were resident in Italy until approximately 500 years ago, thus being a native species in this country (Salari et al. 2020). Since 2017–2019, Eurasian beavers have been found suddenly reappeared, possibly following unofficial releases, also in Central Italy, where they established widespread reproductive populations in two regions, Tuscany and Umbria (Pucci et al. 2021; Mori et al. 2021b, 2022; Viviano et al. 2022). In these areas of Central Italy, another semi-aquatic large-sized rodent, the coypu *Myocastor coypus*, an alien invasive species of South-American origin, is present following introductions for fur-farming; their populations have been expanding since the 1960s (Schertler et al. 2020; Mori et al. 2022). Afterwards, beavers were also detected in other Central-Southern Italian regions (i.e., Abruzzi, Molise and Campania: Capobianco et al. 2023).

In 2022, Italian Administrations and the Italian Mammal Society recommended the removal of beaver individuals from Central Italy (https://www.mammiferi.org/pubblicazioni/posizione-ufficiale-di-atit-sulla-gestione-dei-nuclei-di-castori-eurasiatici-in-centro-italia/ Accessed on 15.02.2023 [only in Italian]), as they most likely resulted from an illegal release or an escape from captivity. However, the Eurasian beaver is listed in Annex IV of the EU Habitats Directive (1992/43/EC, Annex IV: “species requiring a strict protection regime across their entire natural range within the EU, both within and outside Natura 2000 sites”). Also, other beaver populations which have resulted from illegal releases apart from Italy (i.e., those in Belgium, Spain and Scotland) have been permitted to remain, especially after public reaction (e.gDewas et al. 2012; Parker et al. 2012; Crowley et al. 2017; Coz and Young 2020). Thus, its recent naturalisation in Italy may legally prevent any removal action and impose tight population monitoring. As to Italy, individuals from the North-Eastern regions should be monitored following the requirements of the Habitats Directive (as naturally present following range expansion from Austria: Pontarini et al. 2019), whereas those in Central and Southern Italy have been most likely unofficially released, and, with the permissions in derogation from the EU, they could be treated as non-native species.

Despite the lack of reference samples from relict populations, all newly established populations of Eurasian beavers in Europe are characterized by a high genetic diversity (due to translocations from different areas), which may in turn promote range expansion (Munclinger et al. 2022). Beaver individuals from Central Italy belong to the Western mitochondrial DNA clade, which includes Central and Eastern European populations (cf. Mori et al. 2021b; Pucci et al. 2021).

Human-beaver coexistence have been studied in most European countries (e.g., Nolet and Rosell 1998; Liarsou 2013; Swinnen et al. 2017; Janiszewski and Hanzal 2021). Different countries with similar cultural landscapes may show different public attitudes towards beavers, ranging from negative to positive, which may or may not relate to the local ecology of this species (Curry-Lindahl 1967; Siemer et al. 2013; Auster et al. 2020b, 2022b). Imposing strong management actions (even if beavers have been unofficially released) could trigger a chronic, expensive, and emotionally exhausting problem, if the actions are not publicly supported.

In this work, we seek to explore the perceptions of the public towards the presence of the Eurasian beaver in Central Italy using direct questionnaires, thereby gaining insight into the social factors that will need to be considered by decision-makers.

## Materials and methods

We prepared an exploratory questionnaire to investigate how citizens perceive the presence of the beaver in Central Italy, following the survey methods from Great Britain, so to have a reliable comparison (Auster et al. 2020b).

### Participants and Ethics

We directly surveyed citizens in person, in towns and villages, within the vicinity of sites where the presence of beavers has been identified (i.e., Umbria and Toscana). Our study area was defined by the region in which the beavers have been identified, and residents in this area may have had direct experience with the species. The participants were convenience sampled as this is an early-stage, exploratory study to gain an indicative insight into the attitudes of people who live in the local area (Muboko et al. 2016; Gargioni et al. 2021). Whilst this means numbers cannot be directly inferred to represent the prevalence of opinions in wider populations, the study places emphasis on exploring levels of support for reintroduction among this group in response to participant background variables. Surveys were completed between March and November 2022. Before completing the questionnaire, participants were required to declare that they were over 18 years old and able to fill the questionnaire autonomously. Research information was provided prior to participation, and participants were required to give informed consent to participate in this research following the National and International Italian laws on privacy and sensitive data (DL 196/2003; EU Regulation 2016/679), in line with the informed consent method and laws in Italy (Gargioni et al. 2021; Franchini et al. 2022). The information provided for participants is attached as supporting information (Supplementary Material 1). All questionnaires were submitted anonymously and self-completed; participants were provided with an *ad-hoc* QR code to access the survey in their own time, to avoid potential influences by operators.

### Question design

**Fig. 1:**
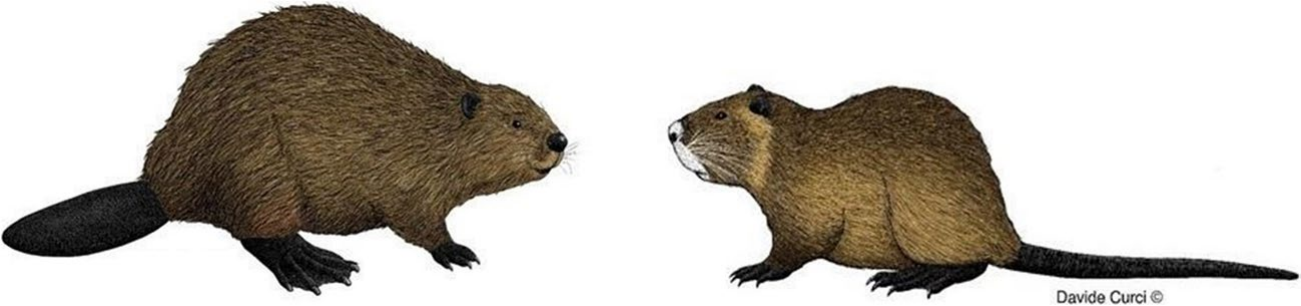
The coloured plate used to test whether citizens were able to distinguish between beavers (on the left) and coypus (on the right)

Questions were informed by a prior survey example (Auster et al. 2020b) and adapted to the local context. They were arranged into three main sections (Supplementary Material 1):

1. Given the local occurrence of non-native coypus *Myocastor coypus*, citizens were firstly asked for their ability to identify the difference between beavers and coypus, by asking them to distinguish between the two species visually (through a coloured plate: Fig. 1) and to identify aspects of their behaviour / signs of presence (two questions).
2. The second part of the questionnaire focused on views of beaver reintroduction and of potential beaver removal from Central Italy. We used multiple choice questions based on a Likert scale, i.e., a scale in which respondents rated their answers from “strongly oppose” (score 1) to “strongly support” (score 5), following Allen and Seaman (2007). Open questions were added to explain the reason for their answers. A further open question asked about potential future impacts by reintroduced / released beavers. Open answers were then classified by dividing them according to their main point(s) (Supplementary Material 2).
3. The third and last part of the questionnaire focused on demographic information. This included: gender; occupation; and location (i.e., Italian region in which participants lived).

The questionnaire text is available in the supporting information (Supplementary Material 1). Questionnaires took 10–15 min to be completed.

### Analysis

We excluded blank and irrelevant (i.e., unlinked to the question, or nonsense) answers from our analyses. All analyses were performed on IBM SPSS Statistics 23 × 64 and R version 4.2.2 (R Core Team 2022), packages *ggplot2* (Wickham et al. 2016) and *ordinal* (Christensen 2018). We conducted ordinal regression analyses to test whether background variables affected views on beaver releases and removal (assessed through the Likert scale, with 5 levels: Supplementary Material 2). Proportional odds regression is used when more than two outcome categories are ranked in an order (Brant 1990). The most important underlying assumption, named as “proportional odds assumption” is that no input variable has a disproportionate effect on a specific level of the response variable. Furthermore, the dependent variable should be measured on an ordinal level, whereas independent variables may be continuous, categorical or ordinal. These assumptions were fulfilled in our dataset (Brant 1990). The main advantage of this method is that the regression parameters have the simple and useful odds ratio interpretation. No multi-collinearity was detected in our dataset, i.e., independent variables were never highly correlated with each other. We kept “Students” as the reference category for each model, as being the largest group (Supplementary Material 2). In particular, we also considered “Students” as a reference, because most of them were attending Biological or Natural Science courses at the University (as they autonomously reported in the questionnaire). Therefore, they were considered all at the same level of general knowledge on conservation biology, thus providing a reliable reference class (cf. Brant 1990). Each variable (i.e., occupation category) was used in a single model to create binary variables with respect to the reference category: 0—student, 1 – each other occupation. Odds ratio values and 95% confidence intervals were used to measure the association between the variable and the outcome (Brant 1990). In these models, R^2^ cannot be applied as a measure for goodness of fit, as the outcome variable is nominal; therefore, we estimated the Nagelkerke Pseudo-R^2^ values (Smith and McKenna 2013).

We tested the level of support for reintroduction in response to an indicative “Level of Knowledge” score, identified from answers to multiple choice questions on the ecology of Eurasian beavers (Supplementary Material 1). Each correct answer scored 1 point. We obtained a total score which was assigned to a “Level of Knowledge” category: 0 = “No Knowledge”; 1 or 2 = “Little Knowledge”; 3 = “Good Knowledge”. We applied chi‐square tests of independence on multiple response sets to test relationships between support to beaver reintroduction and opposition to beaver removal, as well as between these answers and the perceived impact by each respondent. Traditional Pearson chi-square tests cannot be used for multiple response questions, as data in contingency tables are not mutually exclusive; thus, we used and adjusted test as a proxy for marginal associations (Thomas and Decady 2004). The Pearson chi-square test was also used to test whether respondents supporting beaver reintroduction and the response variable on which impact may trigger in the future the population of the Eurasian beaver in Central Italy.

Occupations of respondents were grouped in 23 categories (Supplementary Material 2), including at least 10 respondents each. Occupations declared by less than 10 respondents (i.e., less than 1% respondents) were grouped in the category “Other” (e.g., Huff and Tingley 2015; Auster et al. 2020b). Typologies of occupations and categories of open answers are summarized in Supplementary Material 2.

## Results

**Fig. 2:**
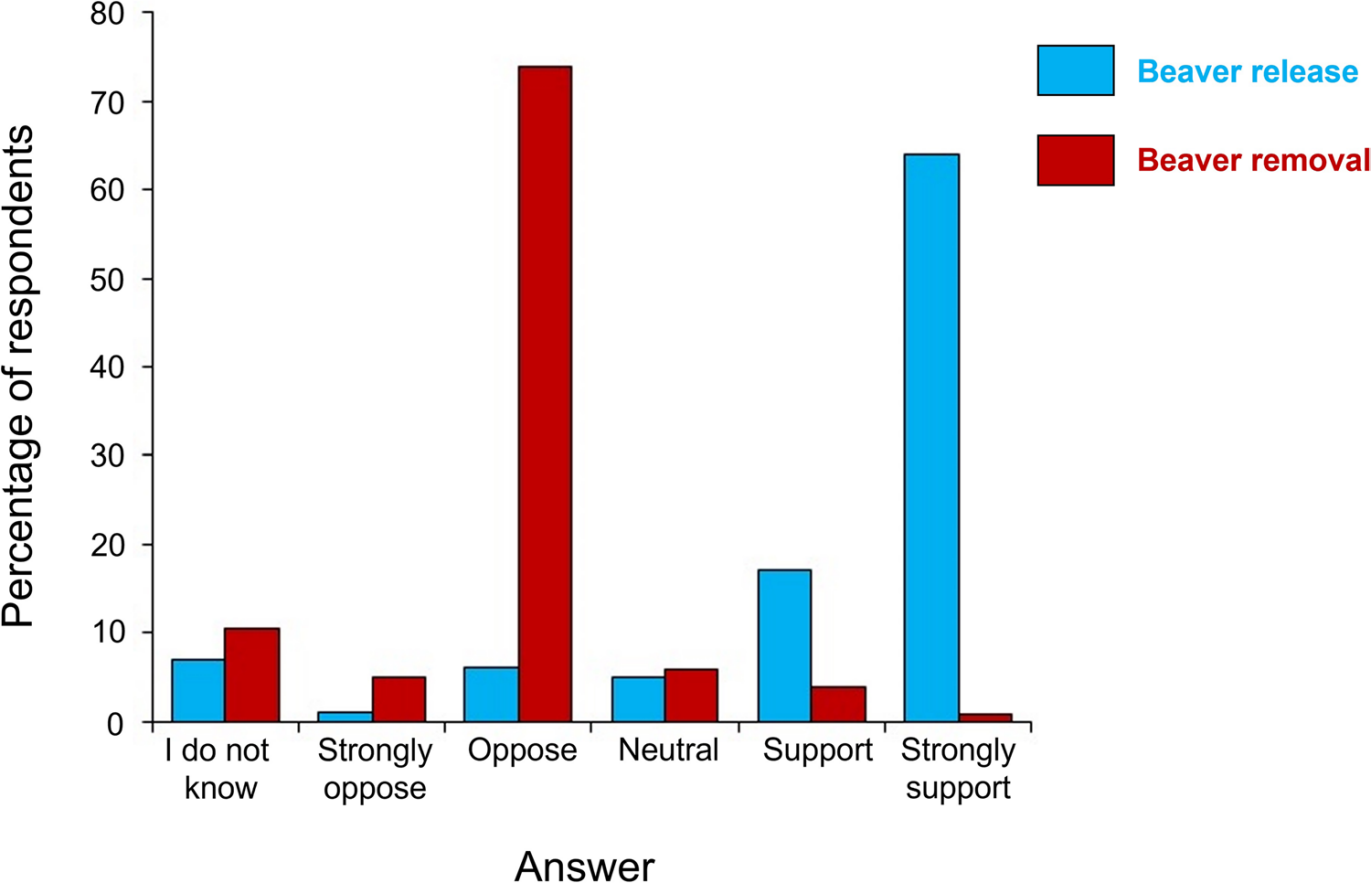
Percentage of answers (*N* = 1114 for each question) in relation to beaver releases (blue bars) and removal (red bars) from Central Italy, classified following the five levels of the Likert scale

We collected a total of 1114 questionnaires (46% women, 52% men, and 2% identified with another gender, Supplementary Material 2), most of them by residents in Central Italy (i.e., 31.6% from Tuscany and 13.5% from Umbria, Supplementary Material 2). Most respondents (94.8%, Supplementary Material 2) were aware of the presence of beavers in Italy and they got the three questions on beaver/coypu ID questions correct. The remaining 5.2% respondents were unaware of the presence of the Eurasian beaver in Italy and provided wrong answers. As to knowledge on beaver presence/ecology, only 4.9% respondents declared no knowledge, 23.2% little knowledge, and 71.9% good knowledge (Supplementary Material 2). Table 1 provides the outcomes of the ordinal regression analysis, demonstrating which occupations were identified as more or less likely to have a more positive view on beaver releases. Occupational groups with the highest number of respondents were “Students” (21.0%, Supplementary Material 2) and personnel employed in “Environment, Nature & Wildlife” sector (21.0%). Respondents with “No Knowledge” or “Little Knowledge” on beaver ecology were less likely than those with “Good Knowledge” to have a supportive view on whether to reintroduce beavers. Respondents with “Little Knowledge” (pooled with “Moderate Knowledge”) were associated with an odds ratio of 0.48 (95% CI, 0.31–0.57; Wald χ^2^ = 5.32, *P* < 0.05) and those with “Good Knowledge” were associated with an odds ratio of 0.52 (95% CI = 0.38–0.61; Wald χ^2^ = 15.4, *P* < 0.01). Thus, the occurrence of "Little Knowledge" was associated with a 52% lower chance of supporting beaver releases than in the case of "Good Knowledge". “Lack of Knowledge” was kept as the reference category (Nagelkerke Pseudo R^2^ = 0.40). Our results showed that 65.5% of the survey respondents strongly supported beaver reintroduction in Italy, whereas only 1.2% strongly opposed it (Fig. 2). Only 3.7% supported the potential of beaver removal from Central Italy (Fig. 2). We observed a highly significant interaction between support for reintroduction and a lack of support for removal operations (χ^2^ = 152.0, df = 2, *P* < 0.001). Among those who were opposed to beaver removal, reasons most commonly given were because of animal right feelings (37.0%: Supplementary Material 2), because beavers are “native/iconic species” (15.5%, Supplementary Material 2), or because they think that a removal project would represent a “waste of time and resources” (11.8%, Supplementary Material 2).

**Table 1:** Ordinal regression analysis and odds ratios, examining support for reintroduction in relation to the occupations of survey participants. Full statistics are reported only for statistically significant results (*P* < 0.05). Students represented the reference category. Participants who identified their occupation as 'Other' specified their occupations as: archaeologist (*N* = 1), carpenter (*N* = 1), chemistry (*N* = 3), counselling (*N* = 3), electrician (*N* = 1), escort (*N* = 4), fashion and marketing (*N* = 3), geologist (*N* = 2), lawyer (*N* = 2) and transport (*N* = 2). Nagelkerke Pseudo R^2^ = 0.33)

		Confidence intervals			
Occupation	Odds ratio	Lower bound	Upper bound	Wald χ^2^	Nagelkerke Pseudo-R^2^
Architecture, Energy & Engineering (*N* = 36)	1.35	0.74	2.56		
Arts, Sport & Media (*N* = 23)	1.19	0.91	1.58		
Building & Maintenance (*N* = 11)	1.31	0.67	2.00		
Business & Finance (*N* = 23)	1.97	1.22	2.35		
Community & Social Service (*N* = 25)	1.13	0.41	1.73		
Computer & Mathematical (*N* = 29)	1.10	0.40	1.69		
Education (*N* = 94)	1.04	0.67	1.69		
Environment, Nature & Wildlife (*N* = 236)	1.60	0.87	2.83	5.41	0.02
Farming & Agriculture (*N* = 29)	1.76	0.87	2.83	4.23	0.04
Fisheries & Aquaculture (*N* = 36)	0.58	0.47	0.77	4.99	0.02
Forestry & Woodland Management (*N* = 17)	1.98	1.28	2.48		
Healthcare (*N* = 59)	0.80	0.45	1.17		
Hospitality (*N* = 15)	0.85	0.56	2.36		
Office & Administrative Support (*N* = 24)	0.71	0.32	1.71		
Other (*N* = 22)	1.26	0.84	1.62		
Physical & Social Sciences (*N* = 10)	0.64	0.32	2.03		
Production (*N* = 28)	1.22	0.68	2.09		
Public Administration (*N* = 51)	1.20	0.65	2.11		
Retired (*N* = 34)	1.46	1.22	1.79		
Sales (*N* = 28)	1.48	1.32	3.70		
Scientific Research (*N* = 11)	1.78	1.08	2.99		
Student (*N* = 238)	0.93	0.68	1.45		
Tourism (*N* = 22)	1.86	1.15	2.54

**Fig. 3:**
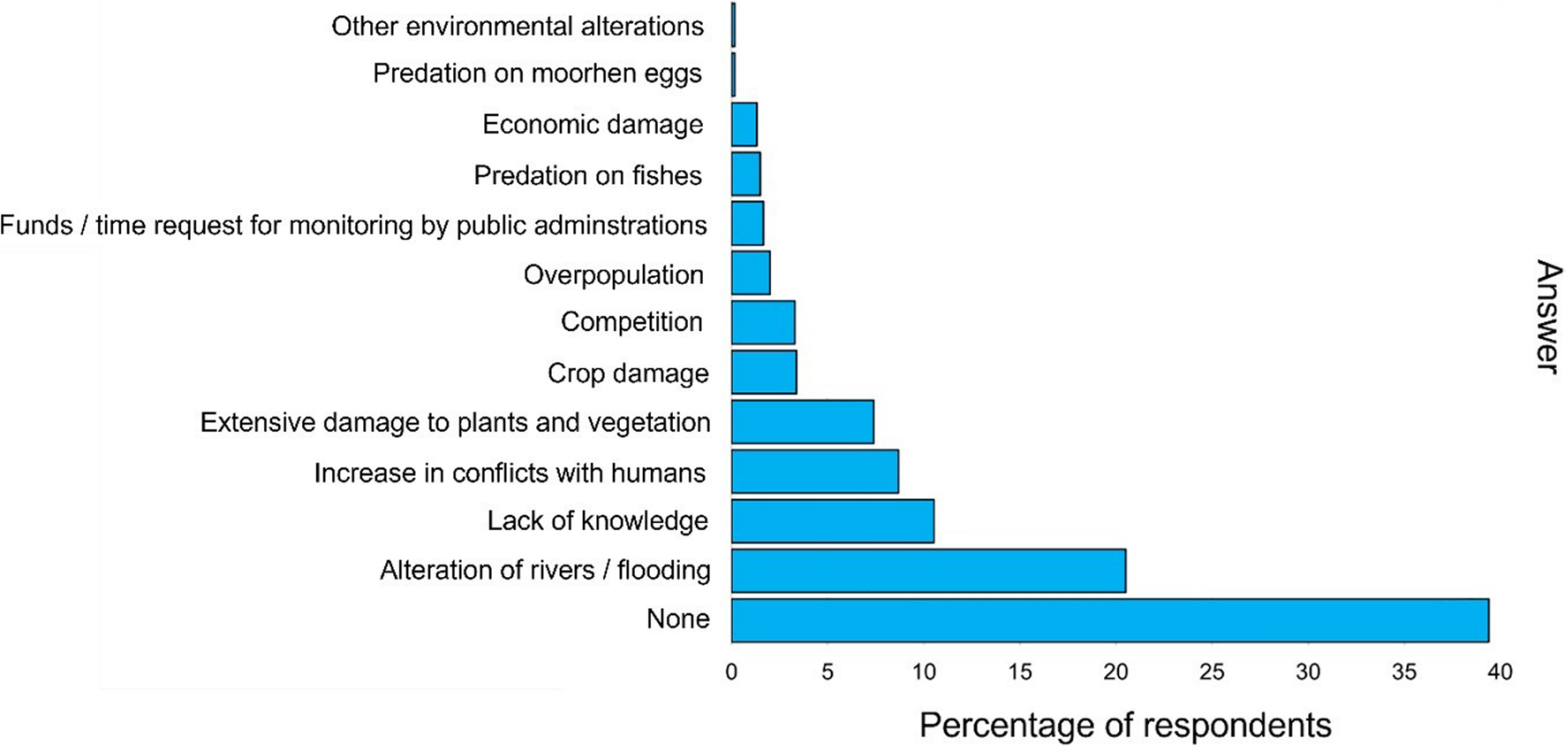
Percentages of answers on potential future impacts by Eurasian beaver as perceived by the surveyed human population in Central Italy

When asked which potential future impacts beaver presence may trigger, most respondents perceived that there will be no impact (Fig. 3). We observed a strong interaction between support for reintroduction and a perception of no future impact (χ^2^ = 44.1, df = 2, *P* < 0.001). However, 20.5% respondents considered that the presence of beaver could result in future alteration of rivers and flooding (Supplementary Material 2; Fig. 3).

## Discussion

Human perception on wildlife releases and management needs full consideration in biodiversity and conservation programs, as it may influence the success of any action (Estévez et al. 2015; Kapitza et al. 2019). The identification of potential stakeholders is pivotal in decision-making systems, particularly when concerning species influencing ecosystem services and functions, e.g., beavers. In our survey, we observed that respondents whose occupation was related to “Fisheries & Aquaculture” were less likely to be supportive of the presence and of reintroduction of beavers in Central Italy. Conversely, we surveyed people employed in “Farming & Agriculture” and “Environment, Nature, and Wildlife” were more likely to be positive towards beaver reintroduction. Actually, it is interesting to note that the “Farming & Agriculture” respondents were more likely to be positive towards beaver reintroduction in participant groups as, in other parts of Europe, varied responses to beaver reintroduction have been observed within this occupation sector (Auster et al. 2020b; Ulicsni et al. 2020). As this was an exploratory perception study, we recommend further research to understand whether this finding is a result of the convenience sampling approach, or whether there may be features in the farming and agricultural context within this setting that influence these more favorable views.

Despite their morphological and habitat similarity, most respondents were aware of the differences between the coypu, a South American alien rodent present in Central Italy for about 50 years, and the recently arrived Eurasian beaver. It is a possibility this may relate to media coverage of the sudden reappearance of the Eurasian beavers in Central Italy regions in April 2021; the arrival of beavers triggered an impressive media campaign with over 30 newspaper/tabloid magazine articles, so information on this “charismatic” rodent was readily available (Pucci et al. 2023). However, we suggest there may be scope for further exploration of factors that may contribute towards or reduce the ability of individuals to distinguish between these two species.

Most surveyed people identified impacts by introduced coypus, but also recognized the usefulness of the beaver for the ecosystem and its importance for riparian/ wetland management. Although it seems that beavers in Central Italy most likely arrived following unauthorised releases or escapes, over 65% of the respondents supported any beaver reintroduction program in Central Italy. The remaining 35% was composed of respondents unaware of the effect of beaver releases and only 1.2% was of participants opposed it. Most respondents who supported reintroduction cited ecosystem services and benefits to biodiversity that beaver may provide (e.g., Brazier et al. 2021; Thompson et al. 2021; Viviano et al. 2022) in their reasoning, or alternatively because they represent a native species – differently from coypus –deserving of local conservation measures. Similar answers were given in opposition to any removal program from Central Italy. Opposers to beaver removal also claimed that trying to remove beavers may represent a waste of time and resources. Amongst Italian mammals, 16.8% are alien species (on a total of *N* = 125 species: Loy et al. 2019), requiring numerical control or eradication. Respondents suggested that, in time of economic crisis, it would be better to invest funds to remove the alien mammal component (e.g., the northern raccoon *Procyon lotor* and coypu), rather than on removal of Eurasian beavers. Moreover, the Spanish case suggests that beaver removal even following illegal releases is complicated and may be unsuccessful, resulting in wasted resource (Mori et al. 2021b; Calderón et al. 2022; González‐Calderón et al. 2023). Accordingly, in Southern Europe, apart from some extreme animal-right groups, a general consensus and awareness of the impacts of biological invasions seems to occur in the general public (e.g., in the case of grey squirrels *Sciurus carolinensis,* Siberian chipmunks *Eutamias sibiricus* and free-ranging llamas *Lama glama*: La Morgia et al. 2017; Lioy et al. 2019; Cerri et al. 2020; Gargioni et al. 2021). Similarly, charismatic fauna e.g., domestic rabbits *Oryctolagus cuniculus domesticus* (Sogliani et al. 2021), brown bear *Ursus arctos* (Glikman et al. 2019), loggerhead turtles *Caretta caretta* (Jones et al. 2011), and beavers (this work) mostly elicit positive attitudes in the general public.

We observed a very limited opposition to beaver reintroduction in this participant group, and this was linked to a general opposition to the removal of beavers already present in Central Italy Amongst opposers to beaver releases, most respondents suggested that the reintroduction of beavers may bring no benefit or may be harmful to riparian woodland and biodiversity (cf. Supplementary Material 2). Furthermore, despite being aware of historical presence of Eurasian beavers in Central Italy (Salari et al. 2020), opposers claimed that current environmental suitability could be low for beavers. Particularly, little knowledge on beaver ecology significantly increased negative attitudes towards reintroduction efforts and / or positive attitudes towards their removal. In several cases concerning scientific researchers or professors from our respondents (i.e., occupation category: “Scientific Research”), removal of beavers was supported as the beaver releases in Central Italy was most likely to have been unofficially conducted (i.e., with no legal authorization). Therefore, if without legal consequences, this faunistic operation could constitute a precedent that could encourage further releases of species once present in the Italian peninsula. Beaver populations in the region are still far from croplands, and mostly located in very natural areas or at the border with urban areas (Mori et al. 2022), thus limiting the possibility for economic impacts on crops at present (Mikulka et al. 2020). However, our results may also suggest a low social awareness on potential beaver impacts. As the beaver population grows, the negative impacts on crops may increase. In other countries that have done reintroductions, beaver populations of over 100,000 individuals cause major crop problems, requesting compensation to farmers (Janiszewski and Hermanowska 2019; Oliveira et al. 2023).

Accordingly, when we asked about potential future impacts by beavers in Central Italy, although most respondents answered “None” or “Lack of Knowledge”, several others reported potential alteration to rivers (including flooding), as well as conflicts with human activities and wellness, crop damage, and competition with other species.

Eurasian beavers have undergone a severe range decline between Medieval times and early 1900 (Halley and Rosell 2002). Afterwards, several authorized and unauthorised releases have occurred throughout Europe, bringing this species out of the brink of extinction (Halley et al. 2021). Wherever released (Italy included), beavers elicited contrasting feelings in human populations (Auster et al. 2020b; Ulicsni et al. 2020), but mostly oriented towards positive effects by the presence of this rodent, including benefits to ecosystems and improved river flowing (Brazier et al. 2021). However, in Central Italy, few survey participants perceived beavers as a disrupting factor for local ecosystems, and dissemination campaigns may further increase the local awareness on the ecology and behaviour of this rodent (cfr. Jiménez et al. 2015; Mea et al. 2016). Besides reintroduction efforts, no European country has eradicated beavers, regardless of whether they were legally or illegally released. Beaver removal typically requires significant economic investment or incentives; therefore, it could be a valuable option to consider developing coexistence strategies to limit human-wildlife conflicts (Mori et al. 2021b; Calderòn et al. 2022).

To conclude, our results showed that there is a widespread knowledge on beaver ecology in our study area, despite several doubts still occur. Well-addressed informative campaigns for the general public involving human riparian land-use, linked with ecosystem services (e.g., stakeholders involved in fishing, farming, and agricultural practices) may be beneficial and help to answer potential doubts and requirements.

Our work, although exploratory, summarized that beavers have been welcomed in Central Italy by the vast majority of surveyed people. Conversely, the Environmental Ministry and the Italian Mammal Society are asking Central Italian regions to conduct a rapid removal of beaver individuals, as they have been unofficially, thus illegally released. Usually, management actions opposed by the general public turn out to be ineffective (Parker and Murphy 2003; Oppel et al. 2011; Gargioni et al. 2021), besides requiring high costs in terms of funds and human efforts (La Morgia et al. 2017; Robertson et al. 2017). This is particularly evident for charismatic species, e.g. mammals (Lioy et al. 2019; De Groot et al. 2020). Thus, given the wide appreciation towards beavers, any management action should consider the local perception to improve its effectiveness and to limit fund wasting (Cagnacci et al. 2012).

## Supplementary Information

Below is the link to the electronic supplementary material.

Supplementary file1 (DOCX 277 KB)

Supplementary file2 (DOCX 124 KB)
