# Supplementary material for: Eurasian beavers in Central Italy: perceptions in the local community
Source: Naturwissenschaften. 2023 Jun 22;110(4):30. doi: 10.1007/s00114-023-01860-x (PMC10287781; doi:10.1007/s00114-023-01860-x)
Supplement: Supplementary file 1 — Supplementary file1 (DOCX 277 KB) [file 114_2023_1860_MOESM1_ESM.docx]

**Supplementary Material 1**

**Participants’ Ethical Consent Form**

Aim of our research project was to assess the perception of potential stakeholders (fishermen, farmers, but also tourists and local citizens) towards the presence of the Eurasian beaver in Central Italy through a direct, anonymous questionnaire. Perceptions by the general public are useful to wildlife managers to design appropriate and successful management actions. Therefore, also your opinion is important; please help us in this important research project.

**Please note the following ethical and data protection statements prior to participation.**

- Taking part is entirely voluntary, and you may choose to withdraw at any time.
- Participation in this research is anonymous.
- You will not be expected to provide any personal information. In case you decide to provide personal information, these data will be treated as confidential, and will not be used in any way for the personal identification of the participant.
- Our research refers to Central Italy.
- The results from this research will be reported in international scientific papers, and it may be reported on for the end of the ‘Rivers with Beavers’ project and relevant conferences.
- Data will be stored anonymously at the National Research Council in Sesto Fiorentino (Florence). If used in an academic paper, it may be stored in a public repository to make it available to other researchers in line with current data sharing practices.
- This study is funded by the Beaver Trust UK, in collaboration with the Italian National Research Council.
- Provided answers will be used in compliance with the provisions of the GDPR 2016/679.
- We greatly value your involvement in this study. Thank you for any time or contribution which you may be able to make. For further information, please email [emiliano.mori@cnr.it](mailto:emiliano.mori@cnr.it)

| Please tick this box to indicate you have read the ethical and data protection statements and understand that participation is voluntary and anonymous. |  |
| --- | --- |
| Please tick this box if you give your consent to the recording of your interview (for the purposes of transcription only). |  |

**Questionnaire**

*[Tick box – select one or more answers] Beaver vs. coypu*

Do you know these species?


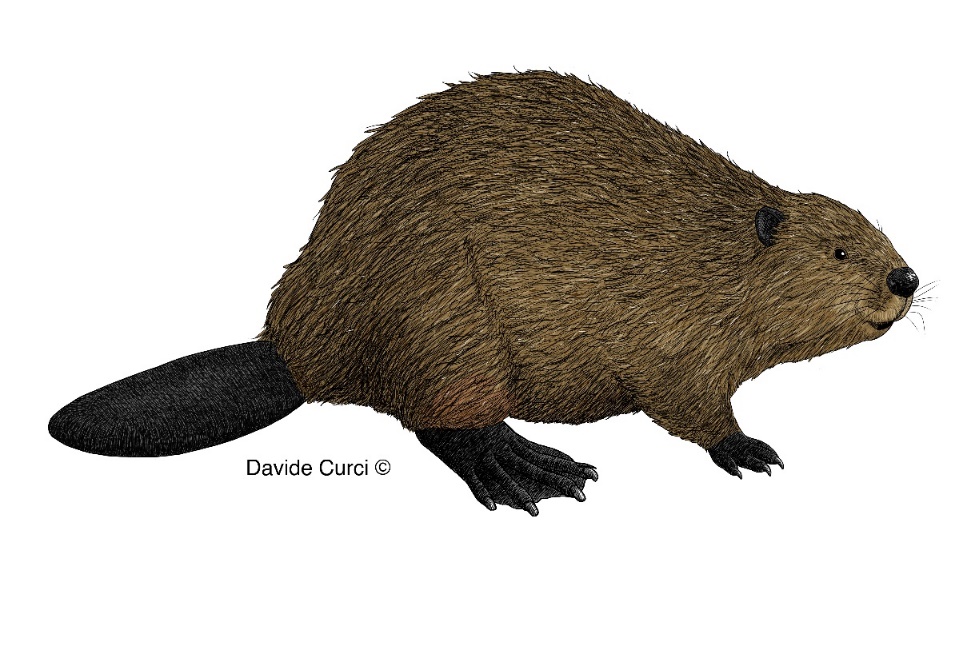

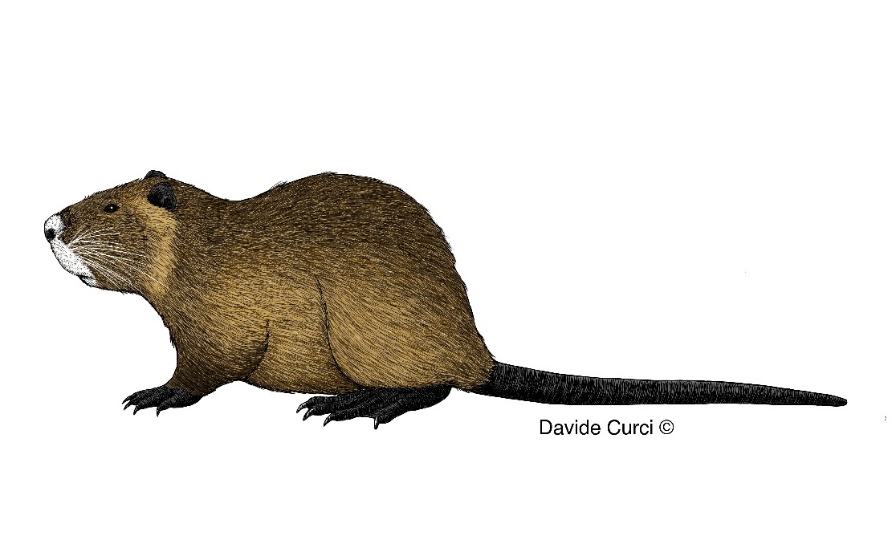


|  | *Beaver* | *Coypu* | *Both Beaver and Coypu* | *I don’t know* |
| --- | --- | --- | --- | --- |
| *Builds dam structures* |  |  |  |  |
| *Feeds on vegetation* |  |  |  |  |
| *Is native to Italy* |  |  |  |  |
| *Causes burrowing* |  |  |  |  |

*[Tick box – select one answer] Only beaver* Prior to this questionnaire, were you familiar with the beaver?

Yes, I have a strong level of knowledge about this species.

Yes, I have a moderate level of knowledge about this species.

Yes, I feel that I have a limited level of knowledge about this species.

No, I had not heard about this species before now.

*[Tick box – select one answer] Only beaver* Prior to this questionnaire, were you aware that this species occurs in Italy?

Yes

No

*[Tick box – select one answer]* With your current level of knowledge, would you support the reintroduction of beaver to Italy?

Yes, I would strongly support reintroduction of this species

Yes, I would support reintroduction of this species

Neutral / No opinion

No, I oppose reintroduction of this species

No, I strongly oppose reintroduction of this species

I don’t know

*[Text box - word limit of 200 words?]* Please **briefly** describe the main reason for your answer.

*[Tick box – select one answer]* A small number of beavers have been identified to be living wild in Central Italy. It is a possibility that they might be removed from the river by public administrations. Would you support or oppose removal of these beavers?

Support

Neutral

Oppose

I don’t know

*[Text box - word limit of 200 words?]* Please **briefly** describe the main reason for your answer.

*[Text box - word limit of 200 words?]* Please **briefly** tell us about any management issues to be considered if this species were to be reintroduced to Italy.

*[Final questions – to be displayed separately following the species-specific questions]*

Finally, please answer the following questions to help us with the analysis for this study. *As a reminder, these are voluntary questions and you may choose to leave them blank.*

When you have finished, please make sure you click ‘Submit’ at the end of this page to ensure your answers have been saved and recorded.

*[Drop-down menu]* How would you describe your primary occupation?

Architecture, Energy & Engineering

Arts, Sport & Media

Building & Maintenance

Business & Finance

Chemistry

Community & Social Service

Computer & Mathematical

Education

Environment, Nature & Wildlife

Farming & Agriculture

Fashion & Marketing

Fisheries & Aquaculture

Forestry & Woodland Management

Healthcare

Hospitality

Lawyer

Office and Administrative Support

Physical and Social Science

Production

Public Adminstration

Retired

Sales

Scientific Research

Student

Tourism

Transport

Other (please specify) *[Text field]*

*[Check box – select one answer]* What is your sex?

Male

Female

Other

*[Check box – select one answer]* May we ask you to identify your age group?

66 or Over

56-65

46-55

36-45

26-35

18-25

*[Thank you screen to be displayed following submission of a completed questionnaire]*

Thank you for taking the time to complete this questionnaire. We are grateful for your contribution. If you chose to leave your contact email address, we will share the final report with you at the time of publication.

Should you have any further questions about this study, please email emiliano.mori@cnr.it
