# Supplementary material for: Eurasian beavers in Central Italy: perceptions in the local community
Source: Naturwissenschaften. 2023 Jun 22;110(4):30. doi: 10.1007/s00114-023-01860-x (PMC10287781; doi:10.1007/s00114-023-01860-x)
Supplement: Supplementary file 2 — Supplementary file2 (DOCX 124 KB) [file 114_2023_1860_MOESM2_ESM.docx]

**Supplementary Material 2**

**Occupation of respondents**

**
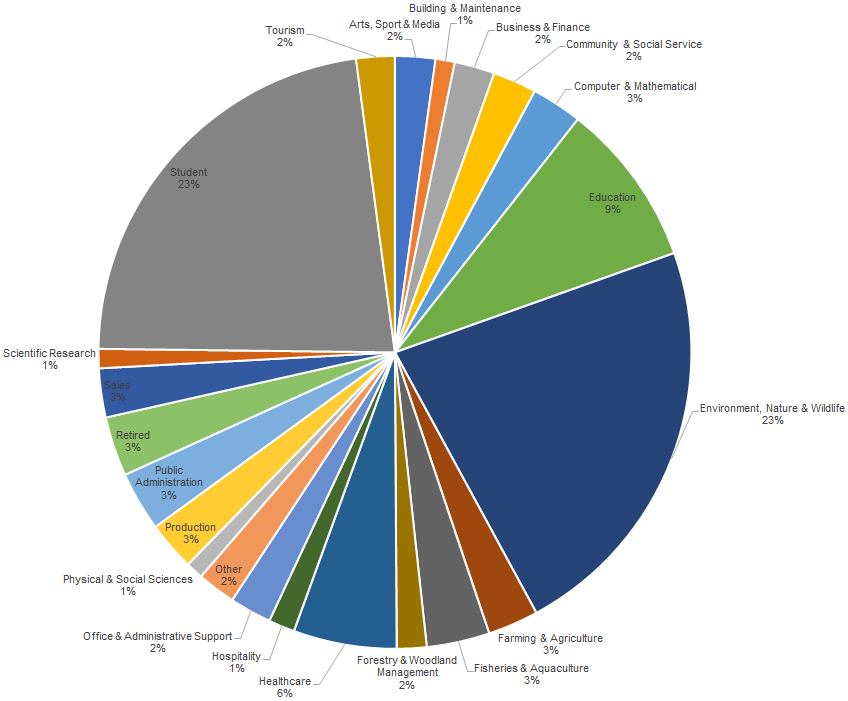
**

**Figure S1.** Occupation of respondents to our survey. Participants who identified their occupation as 'Other' specified their occupations as: archaeologist (N=1), carpenter (N=1), chemistry (N=3), counselling (N=3), electrician (N=1), escort (N=4), fashion and marketing (N=3), geologist (N=2), lawyer (N = 2) and transport (N = 2).

**Classification of open answers**

Open answers are, by definition, different from each other. Therefore, we classified open answers in categories to better show them in our analyses and graphs.

***Question: Would you support the reintroduction of beavers? Please explain why. We classified as “irrelevant” all the comments which did not concern with beavers, their biology and management.***

1. SUPPORT

|  | Reason for support | Further detail | Example quotations | N |
| --- | --- | --- | --- | --- |
| 1 | **General comment of support** | - Yes, definitely!  - Yes, I would support it. | *“They are a nice animal”*  *“All wildlife species need to be supported by conservation measures”* | 56 |
| 2 | **Keystone species** | - Key species | “*It is a keystone species in riverine ecosystems”*  *“They have a disproportionate role in the ecosystems with respect to their local abundance”* | 27 |
| 3 | **Benefits to biodiversity** | - Increase diversity | *“Increased biodiversity benefits all of us”*  *“Beavers benefit local biodiversity, where reintroduced”* | 102 |
| 4 | **Nostalgia** | - Childhood memories  - Experience seeing beavers in wild elsewhere | *“I have seen beaver dams in Poland when I was young”*  *“I am familiar with the species in Denmark”* | 4 |
| 5 | **Already here** | - Reintroduction would be positive as beavers are already occurring in the study area. | “*Beavers are already present in Central Italy; thus, it could be useful to release further individuals to increase local population size”* | 18 |
| 6 | **Ecosystem services** | -Habitat creation  -Ecosystem engineers  -Benefit ecosystem  -Restore natural balance  -Mitigation against climate change | *“Eurasian beavers favour regeneration of local woodlands”*  *“Beavers increase the diversity of macroinvertebrates”*  *“Beavers are helpful in case of droughts”*  *“Beavers create new habitats”* | 194 |
| 7 | **Iconic species** | - Iconic species  - Charismatic | *“Beavers are astonishing and fantastic”*  *“Beavers look like cartoons”* | 52 |
| 8 | **Knowledge of presence elsewhere** | -Already being released elsewhere | *“Beavers have been successfully reintroduced in Austria, Switzerland and they also occur in Northern Italy. They should be released in Central Italy”*  *“Previous reintroduction has been successful (e.g. Austria, Switzerland)”* | 18 |
| 9 | **Ethical motivation** | -Humans eradicated beavers in Medieval times, so they should bring them back  -Moral sense | “*Any animal eradicated by humans in an area should be brought back”*  *“Any wildlife reintroduction counterbalances destructive activities by humans”* | 39 |
| 10 | **Native species** | - Native  - Indigenous  - They are part of the story of our wildlife | *“Our ecosystems coevolved with beavers and they are adapted to this rodent occurrence”*  *“Beavers are a native species in Central Italy and should be replaced whenever possible”* | 189 |
| 11 | **Opportunity for public engagement** | -Tourism  -Engage interest in conservation  -Wildlife enthusiasts | *“Beavers in Mediterranean areas provide an interesting research topic”*  *“Beavers may attract tourists and wildlife photographers along the rivers”*  *“Beavers are iconic and their presence may increase interest and funds for conservation”* | 17 |
| 12 | **Pose little/no threat** | -Cause little problem to human activities (particularly in comparison with coypus and wild boar)  -Don’t see why not | *“As far as I know, no impact by beavers occur where they are present. Other species are more important to be controlled”*  *“No reason they shouldn't be reintroduced”* | 13 |
| 13 | **Concern over feasibility** | - Need to be carefully managed  -Sufficient habitat | *“I would definitely support reintroduction if habitats are still ok for its occurrence”*  *“I would support reintroduction if all stakeholders agree with beaver presence”*  *“I would support it, if potential crop damages are well managed”* | 13 |
| 14 | **Time and resources** | - Good uses of resources | *“Funds for wildlife and for increasing biodiversity are well spent”*  *“A good way to pay young researchers”* | 3 |
| 15 | **Threatened species** | - Threatened species  - Prevent extinction | *“Reintroductions are useful to save rare and threatened species”*  *“Beavers are threatened by habitat loss and reintroduction would prevent their extinction”* | 12 |

1. NEUTRAL/I DON’T KNOW

|  | Reason for neutrality or no opinion | Further detail | Example quotations | N |
| --- | --- | --- | --- | --- |
| 1 | **No benefit** | - Unsure of benefit of reintroduction | *“I do not know if reintroduced beavers would bring any good to local wildlife”*  *“I cannot see any reason for releasing beavers in Central Italy”* | 4 |
| 2 | **Concern over feasibility** | - Unsure of habitat conditions | *“I do not know if current habitats may support beaver releases in Central Italy”* | *12* |
| 2 | **Lack of knowledge** |  | *“I don’t know enough about the pros and cons on beaver reintroductions”*  *“I have to read some more opinion about potential impacts”*  *“I have confused ideas and I have heard about both positive and negative issues”.* | *195* |
| 3 | **Already here** |  | *“They are already here but in small numbers”*  *“They are already present in Italy and they will successfully recolonize without human intervention”* | 2 |
| 4 | **General comment of neutrality** |  | *“I am unsure about impacts. If they are proven to be useful for biodiversity then yes, Eurasian beavers are welcome”* | *26* |
| 5 | **Unknown consequences** |  | *“Consequences of beaver release are unpredictable”* | *51* |

1. OPPOSITION

|  | Reason for opposition | Further detail | Example quotations | N |
| --- | --- | --- | --- | --- |
| 1 | **Waste of time and resources** | - Money spent better elsewhere  - Waste of time | *“It is useless for our life”*  *“Funds rather spent in feeding and housing needy people or to remove invasive species as raccoons and coypus”* | *6* |
| 2 | **Unknown consequences** |  | *“Consequences of beaver release are unpredictable, and it is better to avoid reintroductions”* | *8* |
| 3 | **Concern over feasibility** | - Unsuitable habitat in current time | *“The presence of beaver is not compatible with current strategies of habitat management / human activities”*  *“Beavers may be not compatible with current biodiversity”* | *7* |
| 4 | **Destructive species** | - Destructive species on vegetation  - Predation on eggs and fishes | *“Beavers may prey on fishes and moorhen eggs. Thus, they would have a significant impact on other species and human activities”*  *“I am worried about fish survival in presence of dams and beaver lodges”* | *8* |
| 5 | **General comment of opposition** | - No benefit | *“Reintroduction of beavers brings no benefit and might be harmful to other native or brood species”* | *16* |
| 6 | **Interference with nature** |  | *“Reintroduction is a further human interference with nature and it should be avoided”*  *“Upset of natural equilibrium of river habitats”* | *8* |
| 7 | **Suitability of Central Italy** | - Unsuitable habitat | *“Current rivers in Central Italy are different from those occurring in the Medieval times, when beavers were present”*  *“Central Italy habitats are delicate environments and beavers may bring some species to local extinction”* | *9* |
| 8 | **Already here** |  | *“They are already here but in small numbers, thus it is not important to release them”* | *9* |

***Question: Would you support the removal of beavers from central Italy? Please explain why. We classified as “irrelevant” all the comments which did not concern with beavers, their biology and management.***

1. SUPPORT

|  | Reason for support | Further detail | Example quotations | N |
| --- | --- | --- | --- | --- |
| 1 | **Illegal release** | - Unofficial origins of Eurasian beavers in Central Italy | *“Reintroductions should be guided by laws and precise guidelines including detection of stocks and habitat suitability analyses before releases”*  *“Eurasian beavers were illegally released and they should be removed from Central Italy”* | *24* |
| 2 | **Destructive species** | - Destructive species on vegetation  -Predation on eggs and fishes | *“I am worried about fish survival in presence of dams and beaver lodges”* | *1* |
| 3 | **General comment of support** | -No benefit on their presence | *“There is no benefit in keeping them in the environment”*  *“They could be harmful to other species and should be removed”* | *3* |
| 4 | **Suitability of Central Italy** | - Unsuitable habitat | *“There is not enough wild space for beavers in Central Italy; they would spread into human settlements, gnawing too many trees and creating a damage for crops”* | *1* |

1. NEUTRAL/I DON’T KNOW

|  | Reason for neutrality or no opinion | Further detail | Example quotations | N |
| --- | --- | --- | --- | --- |
| 1 | **Lack of knowledge** |  | *‘I don’t know enough about the pros and cons of beaver removal to have a strong opinion’*  *‘There are few knowledge on beaver ecology and behaviour in Central Italy to support/oppose their removal’* | 125 |
| 2 | **Already here** |  | *“They are already here although in small numbers: it could be useless to remove them”*  *“There have already been records including photographic evidence of Eurasian beavers in Central Italy, so their removal could be useless”* | 3 |
| 3 | **General comment of neutrality** |  | *“I am unsure about implications of beaver removal”* | 36 |
| 4 | **Pose little / no threat** | -Cause little problem to human activities (particularly in comparison with coypus and wild boar) | *“If no impact on human activities and biodiversity is recorded, no need to remove beavers from Central Italy”* | 9 |
| 5 | **Suitability of Central Italy** | - Unsuitable habitat | *“Potentially unsuitable habitats may militate for their local removal”* | 1 |

1. OPPOSITION

|  | Reason for opposition | Further detail | Example quotations | N |
| --- | --- | --- | --- | --- |
| 1 | **General comment of opposition** |  | *“All wild animals are important and should live”*  *“They are so pretty in their habitat, that they should not be removed”* | 413 |
| 2 | **Already here** | - No need to remove them | *“They are here by now, removing them would be stupid”* | 45 |
| 3 | **Ecosystem services** | - Habitat creation  - Ecosystem engineers  - Benefit ecosystem  - Restore natural balance  - Mitigation against climate change | *“Eurasian beavers support the restructuring of native riverine woodland ecosystems”*  *“They may restore pristine biodiversity”*  *“They can help climatic change and summer droughts with their dams”* | 45 |
| 4 | **Ethical motivation** | -Humans eradicated species so why to eradicate also beavers?  -Moral sense | “*Any species eradicated or hunted to extinction should have the right to live in the environment”*  *“Reversing the damage done by human activity”* | 13 |
| 5 | **Native/iconic species** | - Native  - Charismatic  - Iconic | “*Beavers should not be removed as they are native, differently from coypus and raccoons”*  “*Beavers should not be removed as they are iconic”* | 173 |
| 6 | **Opportunity for public engagement** | -Tourism  -Engage interest in conservation/rewilding  -Wildlife enthusiasts | “*The presence of beavers would bring educational opportunities (tourism, naturalist photography, documentary) and interest to the areas they are reintroduced into”* | 1 |
| 7 | **Pose little/no threat** | - Cause little impact  - Low risk for human activities  - Don’t see why not | *“The presence of this species would have no adverse effects on local ecosystems”*  *“No reason they should be removed”* | 68 |
| 8 | **Concern over feasibility** | - Need to be carefully checked  - Sufficient captive places  - Potential social impacts | “*Do we have sufficient space to put them in captivity?”*  *“Are there enough funds to remove them?”*  *“Are we sure about the number of present beavers to be removed?”* | 1 |
| 9 | **Waste of time and resources** | - Wrong uses of resources | “*There are many invasive species in Italy and few funds. Funds should be not employed to remove a native species”*  *“The current population might be quite large, and its removal would require a high amount of funds”*  *“It is impossible to remove the beaver population. Any strong management action would be unsuccessful”* | 132 |
| 10 | **Lack of knowledge** |  | *‘I don’t know enough about the pros and cons to have a strong opinion’*  *‘There are few knowledge on beavers in Central Italy to support/oppose the removal’* | 6 |

***Question: Briefly tell us about any management issues/problems to be considered if this species were to be reintroduced to Italy.***

We classified as “irrelevant” all the comments which did not concern with beavers, their biology and management.

|  | Potential management issue/problem | Further detail / example quotations | N |
| --- | --- | --- | --- |
| 1 | **Alteration of river/flooding** | - Dams/lodge would limit river flowing, often requiring human intervention - Dams would trigger floodings - Beaver excrements would limit river flowing | 236 |
| 2 | **Competition** | - Beavers would outcompete other coexisting species (fish species, large rodents, wild boar, carnivores, coypu) | 38 |
| 3 | **Crop damage** | - Beavers would eat cultivated plants, including vegetables and/or damage orchards by gnawing | 39 |
| 4 | **Human-beaver conflicts** | - Human population would dislike beavers, their presence and their activity; - Public Administration would impose activities along rivers by excluding scientific research or fishing; - Increase of poachers | 100 |
|  | **Extensive damage to plants and vegetation** | - Destruction of natural riverine woodlands; - Impact on vegetal growth | 85 |
| 5 | **Disease transmission** | - Eurasian beavers may bring parasites to native biodiversity - Zoonoses: gonorrhea, ringworm, diarrhea | 5 |
| 6 | **Overpopulation** | - A too large population of beavers should be managed to limit damages to riverine environments, human activities, and disease spread | 23 |
| 7 | **Economic damage** | - Potential conflicts with lumberjacks and reclamation consortium; - Gnawing of electrical cables and pylons | 15 |
| 8 | **Predation on fishes** | - Beavers may kill fishes | 17 |
| 9 | **Predation on moorhen eggs** | - Beavers may consume eggs of the moorhen | 2 |
| 10 | **Funds/time request for monitoring by public administrations** | - Once beavers will be included amongst protected species, funds should be deserved for population monitoring | 19 |
| 11 | **Lack of knowledge** | - No information on management issues/problems | 121 |
| 12 | **None** | - There will be no management issue or problem due to beaver presence | 453 |
| 13 | **Other environmental alterations** | - Presence of beavers will have an effect on local climate | 2 |
